# Comparison of intra-individual coefficients of variation on the paired sampling data when inter-individual variations are different between measures

**DOI:** 10.1186/s13104-016-1912-y

**Published:** 2016-02-19

**Authors:** Fumiaki Kiyomi, Masako Nishikawa, Yoichiro Yoshida, Keita Noda

**Affiliations:** Academia, Industry and Government Collaborative Research Institute of Translational Medicine for Life Innovation, Fukuoka University, 7-45-1 Nanakuma Jyonan-ku, Fukuoka, 814-0180 Japan; Clinical Research Support Center, The Jikei University School of Medicine, 3-25-8 Nishishinbashi Minato-ku, Tokyo, 105-8461 Japan; Department of Gastroenterological Surgery, Fukuoka University Faculty of Medicine, 7-45-1 Nanakuma Jyonan-ku, Fukuoka, 814-0180 Japan; Clinical Research Assist Center, Fukuoka University Hospital, 7-45-1 Nanakuma Jyonan-ku, Fukuoka, 814-0180 Japan

**Keywords:** Intra-individual variation, Inter-individual variation, Coefficient of variation, Mixed effect model, Paired sampling, Bootstrap

## Abstract

**Background:**

Pain intensities of patients are repeatedly measured by Visual Analog Scale (VAS) and Pain Vision (PV) in a clinical research. Two measurements by VAS and PV are performed at the same time. In order to evaluate within patient consistency, intra-individual coefficient of variations (CVs) are compared between measures assuming that the pain status of each patient is stable during the research period. The correlated samples and different inter-individual variation due to different scales of the measures should be taken into account in statistical analysis. The adjustment of covariates will improve the estimation of population mean values of the measures.

**Methods:**

In this paper, statistical approach to compare the intra-individual CVs is proposed. The approach consists of two steps: (1) estimating population mean values and intra-individual variances of the pain intensities by measure in a mixed effect model framework, (2) computing intra-individual CVs and comparing them between measures. The mixed effect model includes measure and some variables as fixed effects and subject by measure as a random effect. The different inter-individual variations between measures and their covariance reflect the paired sampling in the variance component. The confidence interval of the difference of intra-individual CVs is constructed using the asymptotic normality and the delta method. Bootstrap method is available if sample size is small.

**Results:**

The proposed approach is illustrated using pain research data. Measure (VAS and PV), age and sex are included in the model as fixed effects. The confidence intervals of the difference of intra-individual CVs between measures are estimated by the asymptotic theory and by bootstrap using a subgroup resampling, respectively. Both confidence intervals are similar.

**Conclusion:**

The proposed approach is useful to compare two intra-individual CVs taking it into account to reflect the paired sampling, different inter-individual variations between measures and some covariates. Although the inclusion of covariates did not improve the goodness-of-fit in the illustration, the proposed model with covariates will improve the accuracy and/or precision if covariates truly influence response variable. This approach can be applicable with small modification to various situations.

**Electronic supplementary material:**

The online version of this article (doi:10.1186/s13104-016-1912-y) contains supplementary material, which is available to authorized users.

## Background

A clinical research of oxaliplatin chemotherapy was conducted in colorectal cancer patients. Pain intensities of oxaliplatin-induced peripheral neuropathy were assessed by two measures of Visual Analog Scale (VAS) and Pain Vision PS-2100 (PV). The two pain assessments were performed at the same time on the same set of patients and the assessments were repeated during some time interval. VAS is a widely used subjective scale to measure the pain intensity, and patients assess their pain intensities along a continuous line from 0 to 100. PV is an analytical instrument that is designed to assess sense perception and nociception quantitatively and objectively. The pain intensity by PV is defined as a percentage change of two electrical stimulation values with and without pain. Thus measurements by PV are non-negative values (range of the actual data was 0 to about 400). The objective of the research was to compare these subjective and objective measures, and the intra-individual variation was compared in order to evaluate the within patient consistency as the reliability index assuming that the pain status of each patient has been stable during the research period. Since the scales of two measures are different, the intra-individual coefficient of variation (CV) is used as a variation index.

In this situation, there are two conditions to be considered in the statistical analysis:

- correlated samples by paired sampling (assessments by VAS and PV were performed at the same time)
- different inter-individual variation between measures (different scales: 0–100 for VAS and non-negative values of 0 to about 400 for PV)

Furthermore, there exists an additional requirement to improve the estimation of population mean values of measures:

- taking covariates into account, for example sex and/or age

In many clinical studies, the standard deviation, the coefficient of variation or a composite index such as the mean amplitude of glycemic excursion (MAGE) in diabetic area are used as intra-individual variation indexes, and those indexes are calculated for each subject and sometimes compared between groups or treatments by two sample *t* test, linear regression analysis or analysis of variance [e.g. 1–3]. Onishi et al. [4] compared the intra-individual CVs in pre-breakfast self-measured plasma glucose between treatments including some variables as covariates and subject as a random effect. There are many statistical discussions (e.g. 5, 6) relating to comparing intra-individual CVs. Forkman [5] gave a method to compare intra-individual CVs (although “intra-individual” is not explicitly written). Iron [6] compared several methods to calculate intra-class correlation coefficient (ICC). For the paired sampling, Shoukri et al. [7] gave several procedures for testing the equality of two dependent intra-individual CVs. They used a mixed model including one fixed effect and subject as a random effect and they gave a covariance structure reflecting the paired sampling to the model. Their procedures, however, did not clearly model the different inter-individual variation and covariates were not included either. Furthermore, they assumed that the number of repetition of both measurements was the same for all subjects.

In this article, we propose an approach to compare two intra-individual CVs considering the two conditions (correlated samples by paired sampling and different inter-individual variation between measures) and adjustment of the covariates. A mixed effect model will be used to estimate population mean values and intra-individual variances for measures. The mixed effect model includes measure and some variables as fixed effects and subject by measure as a random effect. Different inter-individual variation between measures and the paired sampling are taken into account in this model. The number of repetitions may be varied among subjects due to missing. By using iterative algorithms in the mixed effect models, we obtain the estimates without restriction of the same number of repetitions. The intra-individual CVs will be calculated and compared using these estimates.

## Methods

### Statistical model

Let $y_{ijk} \ge 0$ denote the *kth* observation of the *jth* subject by the *ith* measure, $i = 1,2,\;j = 1,2, \ldots ,n,\;k = 1,2, \ldots ,m_{ij}$. Let $m_{j}$ and $m$ be $m_{j} = m_{1j} + m_{2j}$ and $m = \sum\nolimits_{i,j} {m_{ij} }$. The repeated measurements for the $jth$ subject consist of $m_{1j}$ times examinations by the first measure and $m_{2j}$ times examinations by the second measure. If there are missing values, $m_{1j}$ may be different from $m_{2j}$. Thus, missing values are allowed in the model. In our mixed effect model, $y_{ijk}$ is expressed by

1 $$y_{ijk} = \varvec{x}_{ijk}^{'}\varvec{\beta}+ \gamma_{ij} + e_{ijk} ,$$

where $$\varvec{x}_{ijk}$$ is a $$p \times 1$$ design vector for fixed effects, $$\varvec{\beta}= \left( {\beta_{1} , \ldots ,\beta_{p} } \right)^{'}$$ is a $$p \times 1$$ parameter vector of the fixed effects regression coefficients, $$\gamma_{ij}$$ is the random effect of the $$jth$$ subject for the $$ith$$ measure and assumed to be normally distributed $$\gamma_{ij} \sim N\left( {0, \sigma_{bi}^{2} } \right),$$$$\gamma_{1j}$$ and $$\gamma_{2j}$$ are correlated by a covariance $$\sigma_{b12}^{2}$$ and $$\gamma_{ij}$$ and $$\gamma_{{i^{\prime}j^{\prime}}}$$ are assumed to be independent if $$j \ne j^{'}$$ for $$i, i^{'} = 1,2$$, $$e_{ijk}$$ is the residual error and is assumed that $$e_{ijk} \sim{\text{i}}.{\text{i}}.{\text{d}}.N\left( {0, \sigma_{ei}^{2} } \right)$$. The fixed effect part $$\varvec{x}_{ijk}^{'}\varvec{\beta}$$ includes a factor of the measure, where $$\beta_{1}$$ and $$\beta_{2}$$ denote means for the first measure and for the second measure, respectively.

Using notations of vector, the observations of the $jth$ subject and all observations can be expressed by $\varvec{y}_{j}$ and $\varvec{y}$, where $\varvec{y}_{j} = \left( {y_{1j1} , \ldots , y_{{1jm_{1j} }} ,y_{{2jm_{1j} + 1}} , \ldots ,y_{{2jm_{1j} + m_{2j} }} } \right)^{'}$ and $\varvec{y} = \left( {\varvec{y}_{1}^{\varvec{'}} , \varvec{y}_{2}^{\varvec{'}} , \ldots ,\varvec{y}_{n}^{\varvec{'}} } \right)^{'}$, respectively. Let $\varvec{R}_{j}$ denote a $m_{j} \times m_{j}$ diagonal matrix for the residuals of the $jth$ subject. It can be seen that

$$\varvec{R}_{j} = {\text{diag}}\left( {\underbrace {{\sigma_{e1}^{2} , \ldots ,\sigma_{e1}^{2} }}_{{m_{1j} }},\underbrace {{\sigma_{e2}^{2} , \ldots ,\sigma_{e2}^{2} }}_{{m_{2j} }}} \right).$$

Define $\varvec{ R}$ as ${\text{block diag}} \left( {\varvec{R}_{1} , \ldots ,\varvec{R}_{n} } \right)$. The covariance matrix for the random effect of the $jth$ subject can be expressed by $\varvec{g}$, where

$$\varvec{g} = \left( {\begin{array}{*{20}c} {\sigma_{b1}^{2} } & {\sigma_{b12} } \\ {\sigma_{b12} } & {\sigma_{b2}^{2} } \\ \end{array} } \right).$$

Note that $\varvec{g}$ is the same structure for all $j$. Define the $2n \times 2n$ matrix $\varvec{G}$ as $\varvec{G} = {\text{block diag }}\left( {\varvec{g}, \ldots ,\varvec{ g}} \right)$. In the matrix notation, the model (1) can be represented by

2 $$\varvec{y} = \varvec{X\beta } + \varvec{Z\gamma } + \varvec{e},$$

where $\varvec{X}$ and $\varvec{Z}$ are $m \times p$ and $m \times 2n$ design matrices for the fixed effects and the random effect of subjects, respectively, $\varvec{\gamma}$ is a $2n \times 1$ parameter vector for the random effect and $\varvec{\gamma}\sim N\left( {{\mathbf{0}},\varvec{ G}} \right)$, $\varvec{e}$ is a $m \times 1$ vector of the random residual error and it follows a $N\left( {0, \varvec{R}} \right)$, $\varvec{e}$ and $\varvec{\gamma}$ are independent. $\varvec{\beta}$ is the $p \times 1$ parameter vector mentioned above. We can make $\varvec{X}$ full rank. $\varvec{Z}$ consists of $m \times 1$ column vectors of $\varvec{Z}_{1j}$ and $\varvec{Z}_{2j}$, $j = 1,2, \ldots ,n$, where $\varvec{Z}_{ij} ,i = 1\,2$, has 1 for the corresponding elements to the $jth$ subject by the $ith$ measure and 0 otherwise.

The expectation and the variance of $\varvec{y}$ are expressed by

3 $$E\left( \varvec{y} \right) = \varvec{X\beta }, Var\left( \varvec{y} \right) = \varvec{V} = \varvec{ZGZ}^{'} + \varvec{R}.$$

The components of $\varvec{V}$ are explained in “Appendix 1”.

### Parameter estimation based on likelihood

The parameters in the model (2) are estimated based on the usual maximum likelihood (ML) approach for linear mixed models. The covariance matrix is estimated by the method of restricted maximum likelihood (REML) where degrees of freedom is adjusted in estimating fixed effects [8–11].

According to [11], the log-likelihood can be partitioned into two parts, $L_{1}$ and $L_{2}$. The $L_{1}$ is the part including $\varvec{\beta}$ and $L_{2}$ not including $\varvec{\beta}$. The variance and covariance parameters are estimated using an iterative algorithm based on $L_{2}$, and fixed effect parameters $\varvec{\beta}$ are estimated based on the $L_{1}$ as

4 $$\hat{\varvec{\beta }} = \left( {\varvec{X}^{'} \varvec{V}^{ - 1} \varvec{X}} \right)^{ - 1} \varvec{X}^{'} \varvec{V}^{ - 1} \varvec{y}$$

For the detail of the parameter estimation, see “Appendix 2”.

### Evaluation of the difference of intra-individual CVs

As mentioned in statistical model section, $\beta_{1}$ and $\beta_{2}$ represent means of the first and second measure, respectively. Let $g\left( {\hat{\varvec{a}}} \right)$ denote the difference of CVs between measures, where $\hat{\varvec{a}} = \left( {\hat{\beta }_{1} ,\hat{\beta }_{2} ,\hat{\sigma }_{e1}^{2} ,\hat{\sigma }_{e2}^{2} } \right)^{'}$, $g\left( {\hat{\varvec{a}}} \right) = \sqrt {\hat{\sigma }_{e1}^{2} } /\hat{\beta }_{1} - \sqrt {\hat{\sigma }_{e2}^{2} } /\hat{\beta }_{2}$. Our objective is to test H_0_: $g\left( \varvec{a} \right) = 0$. Using the delta method [12] and $E\left( {\partial^{2} L_{1} /\partial\varvec{\beta}\partial \theta_{l} } \right) = 0$, the expectation and variance of $g\left( {\hat{\varvec{a}}} \right)$ are obtained as

5 $$E\left[ {g\left( {\hat{\varvec{a}}} \right)} \right] \approx \frac{{\sqrt {\sigma_{e1}^{2} } }}{{\beta_{1} }} - \frac{{\sqrt {\sigma_{e2}^{2} } }}{{\beta_{2} }},$$

6 $$\begin{aligned} Var\left[ {g\left( {\hat{\varvec{a}}} \right)} \right] & \approx \frac{1}{{4\beta_{1}^{2} \sigma_{e1}^{2} }}Var\left( {\hat{\sigma }_{e1}^{2} } \right) + \frac{{\sigma_{e1}^{2} }}{{\beta_{1}^{4} }}Var\left( {\hat{\beta }_{1} } \right) + \frac{1}{{4\beta_{2}^{2} \sigma_{e2}^{2} }}Var\left( {\hat{\sigma }_{e2}^{2} } \right) + \frac{{\sigma_{e2}^{2} }}{{\beta_{2}^{4} }}Var\left( {\hat{\beta }_{2} } \right) \\ & + \frac{1}{{4\beta_{1} \beta_{2} \sqrt {\sigma_{e1}^{2} \sigma_{e2}^{2} } }}Cov\left( {\hat{\sigma }_{e1}^{2} ,\hat{\sigma }_{e2}^{2} } \right) + \frac{{\sqrt {\sigma_{e1}^{2} \sigma_{e2}^{2} } }}{{\beta_{1}^{2} \beta_{2}^{2} }}Cov\left( {\hat{\beta }_{1} ,\hat{\beta }_{2} } \right). \\ \end{aligned}$$

Under H_0_, $g\left( {\hat{\varvec{a}}} \right)/\sqrt {\widehat{Var}\left[ {g\left( {\hat{\varvec{a}}} \right)} \right]}$ has the unit normal distribution by applying the large sample theory, where $\widehat{Var}\left[ {g\left( {\hat{\varvec{a}}} \right)} \right]$ is the estimator of $Var\left[ {g\left( {\hat{\varvec{a}}} \right)} \right]$ obtained by substituting estimators for all parameters. Alternatively the equality of CVs between measures can be evaluated by an interval estimation of the difference of CVs using the asymptotic normality of $g\left( {\hat{\varvec{a}}} \right)/\sqrt {\widehat{Var}\left[ {g\left( {\hat{\varvec{a}}} \right)} \right]}$.

Another method to test H_0_ is bootstrap method [13] which is particularly useful if sample size is not sufficiently large to apply the asymptotic theory. A simple bootstrapping is the subject resampling method with replacement. Since the data is obtained based on the paired sampling, the resampling is taken place by subject (kind of block or cluster bootstrap, [14]) in order to capture the dependence structure. The resampled number of subjects is equal to the size $n$ of the original data set. The resampling will be straightforward if the numbers of observations by measure are the same across the subjects, namely $m_{ij} = r_{i} ,\;i = 1,2$ for all $j = 1, \ldots ,n$. Otherwise, a subgroup resampling classified by number of observations by measure will be one way to make the resample data set with the same records $m$ as in the original data set if two measures (e.g. VAS and PV) are always observed paired although the number of observations may be different among subjects. For example, let $n_{1}$, $n_{2}$ and $n_{3}$ ($n_{1} + n_{2} + n_{3} = n$) be defined as the number of subjects repeatedly measured twice, thrice and four times, respectively. Thus there are three subgroups. The resampling is performed in the same size $n_{i}$ with replacement from the $ith$ subgroup $\left( {i = 1, 2, 3} \right)$.

There exist another bootstrap methods such as s parametric bootstrap. Since the objective of this article, however, is not to discuss the bootstrapping, only the blocked and subgrouped way will be used in the later section.

## Application result

Our proposed model was applied to the clinical research data mentioned in the background section. Forty-three patients were treated with oxaliplatin chemotherapy. The pain intensity of oxaliplatin-induced peripheral neuropathy was assessed by two measures of VAS and PV. All patients with repeated measurements were included in the analysis, and the number of records of the data set was $m = 158$ (see Additional file 1). Summary statistics of age and sex and those of PAS and PV were shown in Tables 1 and 2, respectively.

**Table 1:** Summary statistics of age and sex

	Mean (SD)/N (%)^a^	Min–Max
Age	65.4 (8.4)	43–81
Sex		
Female	11 (25.6)	
Male	32 (74.4)	

Sample size: 43 patients

^a^Mean (SD) for age and N (%) for sex

**Table 2:** Summary statistics of the pain intensity data for 43 patients

	VAS	PV
Mean^a^	21.2	26.1
Variance		
Intra^b^	165.7	1725.1
Inter^c^	477.3	274.9
Individual SD^d^		
Mean (SD)	9.2 (8.7)	21.7 (30.7)
Min–Max	0–33	0–172

VAS and PV were repeatedly measured for 43 patients. The number of data records were 158

^a^Calculate mean for each of 43 patients (*m*_subj_), and calculate mean of 43 *m*_subj_ values

^b^Unbiased variance, i.e. $\sum\nolimits_{j} {{\text{SS}}_{j} } /(316 - 43)$, *SS*_*j*_ is a sum of squares for patient *j*

^c^Variance of *m*_subj_

^d^SD for each of 43 patients was calculated

A stopping criterion of numerical iterations by the Fisher’s scoring method to estimate parameters was set to 10^−6^. Sex, age, interaction between measure and sex and interaction between measure and age were included in the mixed model as covariates (fixed effects in full model). To obtain the MLEs of $$\beta_{1}$$$$\left( {\text{VAS}} \right)$$ and $$\beta_{2}$$ (PV) as least square means (LSMs, [15]), let $$\beta_{3}$$, $$\beta_{4}$$, $$\beta_{5}$$ and $$\beta_{6}$$ be regression parameters for sex (0: female, 1: male), interaction between measure and sex (1: VAS and male, 0: otherwise), age and interaction between measure and age (age in year for VAS and 0 for PV). Let $$\bar{x}_{sex}$$ and $$\bar{x}_{age}$$ denote the proportion of male and mean age, respectively. The MLEs of $$\beta_{1}$$ and $$\beta_{2}$$ are $$\varvec{l}_{1}^{ '} \hat{\varvec{\beta }}$$ and $$\varvec{l}_{2}^{ '} \hat{\varvec{\beta }}$$, where $$\varvec{l}_{1} = \left( {\begin{array}{*{20}c} 1 & 0 & {\bar{x}_{sex} } & {\bar{x}_{sex} } & {\bar{x}_{age} } & {\bar{x}_{age} } \\ \end{array} } \right)^{'}$$ and $$\varvec{l}_{2} = \left( {\begin{array}{*{20}c} 0 & 1 & {\bar{x}_{sex} } & 0 & {\bar{x}_{age} } & 0 \\ \end{array} } \right)^{'}$$ and $$\hat{\varvec{\beta }}$$ is obtained by (4). SEs of LSMs are estimated as $${\text{SQRT}}\left( {\varvec{l}_{i}^{'} \left( {\varvec{X}^{'} \hat{\varvec{V}}^{ - 1} \varvec{X}} \right)^{ - 1} \varvec{l}_{i} } \right)$$. Estimated parameters were shown in Table 2.

To obtain variance of $g\left( {\hat{\varvec{a}}} \right)$, $\widehat{Cov}\left( {\hat{\sigma }_{e1}^{2} ,\hat{\sigma }_{e2}^{2} } \right)$ and $\widehat{Cov}\left( {\hat{\beta }_{1} ,\hat{\beta }_{2} } \right)$ should be estimated as shown in (6) in addition to the results in Table 3. Using Fisher’s information matrix we see that $\widehat{Cov}\left( {\hat{\sigma }_{e1}^{2} ,\hat{\sigma }_{e2}^{2} } \right) = - 13.2$ and $\widehat{Cov}\left( {\hat{\beta }_{1} ,\hat{\beta }_{2} } \right) = \varvec{l}_{1}^{'} \left( {\varvec{X}^{'} \hat{\varvec{V}}^{ - 1} \varvec{X}} \right)^{ - 1} \varvec{l}_{2} = 10.0$. The difference of CVs and its 95 % confidence interval was estimated as −0.96 (−1.59, −0.33) by (5) and (6). Thus the intra-individual variation was significantly smaller in the VAS than PV.

**Table 3:** Result of parameter estimation for the pain intensity data

	$\beta_{1}$	$\beta_{2}$	$\sigma_{e1}^{2}$	$\sigma_{e2}^{2}$	$\varvec{ }\sigma_{b1}^{2}$	$\sigma_{b2}^{2}$	$\sigma_{b12}^{2}$
MLE	21.0	26.3	166	1707	474	305	423
SE	3.50	4.25	21.8	220.1	117.6	176.2	120.9

Bootstrapping was also conducted as sensitivity analysis of the asymptotic inference. 1000 data sets with the same size as the original sample were generated with replacement by the subgroup method, and the model parameters and the difference of CVs between measures were estimated for each data set. A Bootstrap estimate of the difference of intra-individual CVs was obtained as the mean of the 1000 estimates, and a 95 % confidence interval was constructed by mean of the 25th and 26th values and mean of the 975th and 976th values out of the 1000 estimates. The bootstrap estimate was −0.90 (−1.50, −0.31), and this estimate was similar as in the asymptotic theory.

### Modeling process and diagnostics

**Fig. 1:**
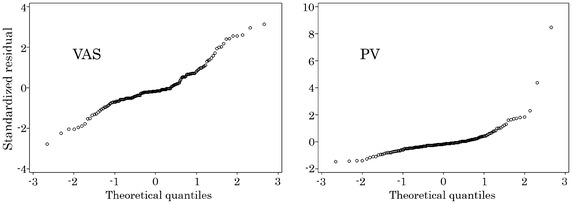
Q–Q plots by measure

The assumption of normality was checked by Q–Q plots (Fig. 1) of residuals. There were several patients whose pain intensities were close to the maximum pain intensity, and the tails on the right side of distributions of VAS and PV were longer, particularly in PV. Since patients with high pain intensities cannot be excluded from the analysis population for our research objective and a logarithmically transformation is not possible for zero values, all patients were included in the statistical analyses. We performed a sensitivity analysis excluding two patients with large values and confirmed similar result as shown in the former analysis.

**Fig. 2:**
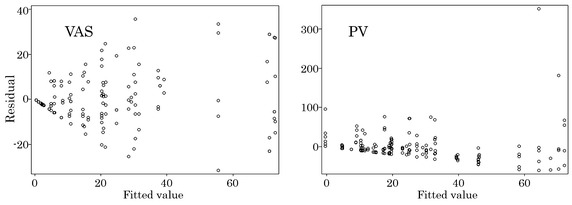
Residual plots by measure

The residual plots for fitted value (Fig. 2) also show several large values in PV and the residual errors do not seem to be totally random. The Q–Q plots and residual plots may suggest to use appropriate data transformations, other distributional assumption and/or robust methods. These methods generally improve model fittings and make comparison of means precise. It is, however, not certain if these methods are relevant to compare intra-individual variations. Therefore, we still assume normality in the analysis. Residual plot for each measure, P–P plot by measure and plot of Cook’s distance against leverage/(1-leverage) are presented for further diagnostic information in Additional file 2: Figures S1, S2 and S3, respectively.

The goodness-of-fit of the full model, where fixed effects were measure, sex, age, interactions between measure and sex and measure and age, was checked in comparison with the null model including only measure as a fixed effect using a likelihood ratio test. The test statistics was 3.048 and P-value according to χ^2^ distribution with four degrees of freedom was 0.550. Thus the addition of sex and age did not contribute to improvement of the model in the pain research data, and the null model will be applied in practice. Since the objective of this application section is to illustrate the availability of the proposed model, we presented the results of the full model assuming normality.

Summary statistics of individual standard deviations (SDs) in VAS and PV were shown in Table 2. The range of individual SDs were vary from 0 to 33 in VAS and from 0 to 172 in PV. The comparison of the intra-individual CV between VAS and PV corresponds to compare the average individual SDs by mean.

## Conclusion

Statistical approach to compare two intra-individual CVs considering the paired sampling, different inter-individual variations between measures and some covariates was proposed. The approach is (1) estimating intra-individual variances and mean values of measures by the mixed effect model including measure and some covariates as fixed effects and subject by measure as a random effect to reflect different inter-individual variation between measures and the paired sampling, (2) computing intra-individual CVs and comparing these CVs between measures. The utility of the approach was shown by applying to the real pain research data. Although the inclusion of covariates did not improve the goodness-of-fit in the illustration, the proposed model with covariates will improve the accuracy and/or precision if covariates truly influence response variable.

The proposed approach is applicable for many situations. One example is to give $\sigma_{b1}^{2} = \sigma_{b2}^{2} = \sigma_{b}^{2}$ if the same inter individual variations can be assumed between measures. In a study [16], the frequently-sampled intravenous glucose tolerance test was carried out for subjects by two protocols in order to estimate insulin sensitivity, and intra-individual CVs of the insulin sensitivity were compared between protocols. If our proposed model is applied to this data, the same inter individual variation ($\sigma_{b1}^{2} = \sigma_{b2}^{2}$) for two protocols will be observed since the same scaled variable was measured by the two different protocols.

Another example is to compare intra-individual variances $\sigma_{ei\left( z \right)}^{2}$ ($i = 1,2$) by analyzing $z_{ijk} = \log \left( {y_{ijk} } \right)$ if lognormal distributions can be assumed for $y_{ijk}$ and the geometric CV [17] of the original data can be approximated by ${\text{SQRT}}\left\{ {\exp \left( {\sigma_{ei\left( z \right)}^{2} } \right) - 1} \right\} \cong \sigma_{ei\left( z \right)}^{2}$, where $\sigma_{ei\left( z \right)}^{2}$ is the residual error variance of $z_{ijk}$ and it is assumed to be small enough to hold the approximation. The approximation of the geometric CV means that CVs in original scale data can be approximated by variances in the logarithmically transformed data. This procedure suggests to omit the CV calculation step. For example, area under the pharmacokinetic curve and triglyceride have such characteristics. These variables have skewed distributions with longer tail upper side, and are frequently logarithmically transformed before statistical analyses. However, note that possible biases should be discussed before the transformation since the logarithmic transformation can give a larger benefit to a group with large values than the other group with small values when estimating population intra-individual variances.

An extension to multi-group comparisons is straightforward by giving corresponding $\varvec{R}_{j}$ and $\varvec{G}_{j}$ and by deriving derivatives of $\varvec{V}$.

## Appendix 1: Components of variance $\varvec{V}$

Let $\varvec{V}_{j} = Var\left( {\varvec{y}_{j} } \right)$ denote the $m_{j} \times m_{j}$ variance matrix of the $jth$ subject. Note that $\varvec{V}_{j} ,j = 1, \ldots ,n,$ have the same structure except the dimension of the repetition $m_{ij} , i = 1,2$. The variance matrix $\varvec{V}$ is given as below.

$$\varvec{V} = {\text{block diag }}\left( {\varvec{V}_{1} , \ldots , \varvec{V}_{n} } \right),$$

where

$$\varvec{V}_{j} = \left( {\begin{array}{*{20}c} {\varvec{A}_{1j} } & {\varvec{B}_{12j} } \\ {\varvec{B}_{12j}^{\varvec{'}} } & {\varvec{A}_{2j} } \\ \end{array} } \right),$$

$$\varvec{A}_{ij} = \left( {\begin{array}{*{20}c} {\sigma_{bi}^{2} + \sigma_{ei}^{2} } & {\sigma_{bi}^{2} } & \cdots & {\sigma_{bi}^{2} } \\ {\sigma_{bi}^{2} } & {\sigma_{bi}^{2} + \sigma_{ei}^{2} } & \cdots & {\sigma_{bi}^{2} } \\ \cdots & \cdots & \cdots & \cdots \\ {\sigma_{bi}^{2} } & {\sigma_{bi}^{2} } & \cdots & {\sigma_{bi}^{2} + \sigma_{ei}^{2} } \\ \end{array} } \right) = \sigma_{bi}^{2} \varvec{J}_{{m_{ij} \times m_{ij} }} + \sigma_{ei}^{2} \varvec{I}_{{m_{ij} }}$$

$$\varvec{B}_{12j} = \left( {\begin{array}{*{20}c} {\sigma_{b12} } & \cdots & {\sigma_{b12} } \\ \cdots & \cdots & \cdots \\ {\sigma_{b12} } & \cdots & {\sigma_{b12} } \\ \end{array} } \right) = \sigma_{b12} \varvec{J}_{{m_{1j} \times m_{2j} }} ,$$

$\varvec{I}_{u}$ is a $u \times u$ unit matrix and ${\mathbf{J}}_{u \times v}$ is a $u \times v$ matrix with all the elements equal to 1.

## Appendix 2: Parameter estimation

According to [11], the log-likelihood can be partitioned into two parts, $L_{1}$ and $L_{2}$, where $L_{1}$ is the part including $\varvec{\beta}$ and $L_{2}$ not including $\varvec{\beta}$. $L_{1}$ and $L_{2}$ are expressed as below.

7 $$\begin{aligned} L_{1} & = - \frac{1}{2}\left[ {p\log 2\pi + \log \left| {(\varvec{X}^{'} \varvec{V}^{ - 1} \varvec{X})^{ - 1} } \right|} \right. \\ & \quad \left.+ \left\{ {(\varvec{X}^{'} \varvec{V}^{ - 1} \varvec{X})^{ - 1} \varvec{X^{\prime}V}^{ - 1} \varvec{y} -\varvec{\beta}} \right\}^{'} (\varvec{X}^{'} \varvec{V}^{ - 1} \varvec{X})\left\{ {(\varvec{X}^{'} \varvec{V}^{ - 1} \varvec{X})^{ - 1} \varvec{X}^{'} \varvec{V}^{ - 1} \varvec{y} -\varvec{\beta}} \right\}\right], \\ \end{aligned}$$

8 $$L_{2} = - \frac{1}{2}\left[ {C + \log \left| \varvec{V} \right| + \log \left| {\varvec{X}^{'} \varvec{V}^{ - 1} \varvec{X}} \right| + \varvec{y}^{'} \varvec{Py}} \right],$$

where $C$ is a certain constant and $\varvec{P} = \varvec{V}^{ - 1} - \varvec{V}^{ - 1} \varvec{X}\left( {\varvec{X}^{'} \varvec{V}^{ - 1} \varvec{X}} \right)^{ - } \varvec{X}^{'} \varvec{V}^{ - 1}.$ The variance and covariance parameters $\varvec{\theta}= \left( {\theta_{1} ,\varvec{ }\theta_{2} ,\varvec{ }\theta_{3} ,\varvec{ }\theta_{4} ,\varvec{ }\theta_{5} } \right)^{\varvec{'}} = \left( {\sigma_{e1}^{2} ,\varvec{ }\sigma_{e2}^{2} ,\varvec{ }\sigma_{b1}^{2} ,\varvec{ }\sigma_{b2}^{2} ,\varvec{ }\sigma_{b12}^{2} } \right)^{\varvec{'}}$ are estimated based on $L_{2}$, while the fixed effect parameters $\varvec{\beta}$ are estimated based on the $L_{1}$. The first order derivatives of $L_{2}$ is

9 $$\frac{{\partial L_{2} }}{{\partial \theta_{l} }} = - \frac{1}{2}\left[ {{\text{tr}}\left( {\varvec{P}\frac{{\partial \varvec{V}}}{{\partial \theta_{l} }}} \right) - \varvec{y}^{'} \varvec{P}\frac{{\partial \varvec{V}}}{{\partial \theta_{l} }}\varvec{Py}} \right].$$

$\partial \varvec{V}/\partial \theta_{l}$ are obtained by simple manipulations as

$$\frac{{\partial \varvec{V}}}{{\partial \sigma_{ei}^{2} }} = {\text{diag }}\left( {\frac{{\partial \varvec{R}_{1} }}{{\partial \sigma_{ei}^{2} }}, \ldots ,\frac{{\partial \varvec{R}_{n} }}{{\partial \sigma_{ei}^{2} }}} \right),\quad \frac{{\partial \varvec{R}_{j} }}{{\partial \sigma_{e1}^{2} }} = {\text{diag }}\left( {1, \ldots ,1,0, \ldots ,0} \right),\quad \frac{{\partial \varvec{R}_{j} }}{{\partial \sigma_{e2}^{2} }} = {\text{diag }}\left( {0, \ldots ,0,1, \ldots ,1} \right)$$

10 $$\frac{{\partial \varvec{V}}}{{\partial \sigma_{b1}^{2} }} = \varvec{Z}\left( {\begin{array}{*{20}c} {\begin{array}{*{20}c} 1 & 0 \\ 0 & 0 \\ \end{array} } & {} & {} \\ {} & \ddots & {} \\ {} & {} & {\begin{array}{*{20}c} 1 & 0 \\ 0 & 0 \\ \end{array} } \\ \end{array} } \right)\varvec{Z}^{'} ,\varvec{ }\quad \frac{{\partial \varvec{V}}}{{\partial \sigma_{b2}^{2} }} = \varvec{Z}\left( {\begin{array}{*{20}c} {\begin{array}{*{20}c} 0 & 0 \\ 0 & 1 \\ \end{array} } & {} & {} \\ {} & \ddots & {} \\ {} & {} & {\begin{array}{*{20}c} 0 & 0 \\ 0 & 1 \\ \end{array} } \\ \end{array} } \right)\varvec{Z}^{'}$$

$$\frac{{\partial \varvec{V}}}{{\partial \sigma_{b12} }} = \varvec{Z}\left( {\begin{array}{*{20}c} {\begin{array}{*{20}c} 0 & 1 \\ 1 & 0 \\ \end{array} } & {} & {} \\ {} & \ddots & {} \\ {} & {} & {\begin{array}{*{20}c} 0 & 1 \\ 1 & 0 \\ \end{array} } \\ \end{array} } \right)\varvec{Z}^{'} .$$

ML estimates $\hat{\varvec{\theta }}$ are the solution of the estimating equations $\partial L_{2} /\partial \theta_{l} = 0$. Usually ML estimates $\hat{\varvec{\theta }}$ are not expressed explicitly and therefore an iterative algorithm such as Newton–Raphson method is used to obtain $\hat{\varvec{\theta }}$. Fisher’s scoring method, in which Fisher’s information matrix ***I***$\left(\varvec{\theta}\right)$ is used instead of Hessian in Newton–Raphson method, is now employed as the iterative algorithm, since $\varvec{I}\left(\varvec{\theta}\right)$ is a simpler form than Hessian in mixed models and the asymptotic variance of $\hat{\varvec{\theta }}$ can be estimated by $\varvec{I}^{ - 1} ( {\hat{\varvec{\theta }}})$ (not observed information [18]). Thus second order derivatives are needed to apply the algorithm

$$\varvec{\theta}_{k + 1} =\varvec{\theta}_{k} + c\varvec{I}^{ - 1} \left( {\varvec{\theta}_{k} } \right)\varvec{S}\left( {\varvec{\theta}_{k} } \right),$$

where $\varvec{\theta}_{k}$ is the $kth$ estimates, $\varvec{\theta}_{k + 1}$ is an improved one, $0 < c < 1$ is a constant value to adjust an improvement quantity and $\varvec{S}\left( {\varvec{\theta}_{k} } \right) = \left( {\partial L_{2} /\partial \theta_{l} } \right)|_{{\varvec{\theta}=\varvec{\theta}_{k} }}$.

Simple manipulations give second order derivatives of $L_{2}$ and $\varvec{I}\left(\varvec{\theta}\right)$, where the $\left( {\theta_{l} , \theta_{{l^{\prime}}} } \right)$ element of the former and the latter as can be expressed as

$$\frac{{\partial^{2} L_{2} }}{{\partial \theta_{l} \partial \theta_{{l^{\prime}}} }} = - \frac{1}{2}\left[ {{\text{tr}}\left( { - \varvec{P}\frac{{\partial \varvec{V}}}{{\partial \theta_{{l^{\prime}}} }}\varvec{P}\frac{{\partial \varvec{V}}}{{\partial \theta_{l} }}} \right) + \varvec{y}^{'} \varvec{P}\frac{{\partial \varvec{V}}}{{\partial \theta_{{l^{\prime}}} }}\varvec{P}\frac{{\partial \varvec{V}}}{{\partial \theta_{l} }}\varvec{Py} + \varvec{y}^{'} \varvec{P}\frac{{\partial \varvec{V}}}{{\partial \theta_{l} }}\varvec{P}\frac{{\partial \varvec{V}}}{{\partial \theta_{{l^{\prime}}} }}\varvec{Py}} \right],$$

11 $$E\left( { - \frac{{\partial^{2} L_{2} }}{{\partial \theta_{l} \partial \theta_{{l^{\prime}}} }}} \right) = \frac{1}{2}{\text{tr}}\left( { - \varvec{P}\frac{{\partial \varvec{V}}}{{\partial \theta_{{l^{\prime}}} }}\varvec{P}\frac{{\partial \varvec{V}}}{{\partial \theta_{l} }}} \right),\varvec{ } l, l^{'} = 1,2, \ldots ,5,$$

respectively. With respect to $\varvec{\beta}$, the ML estimator is derived as the solution of the first order derivatives of (7) equal to 0 as

12 $$\hat{\varvec{\beta }} = \left( {\varvec{X}^{'} \varvec{V}^{ - 1} \varvec{X}} \right)^{ - 1} \varvec{X}^{'} \varvec{V}^{ - 1} \varvec{y}$$

$\varvec{\beta}$ is actually estimated by substituting $\hat{\varvec{V}}$ for $\varvec{V}$ in (13) as $\hat{\varvec{\beta }} = \left( {\varvec{X}^{'} \hat{\varvec{V}}^{ - 1} \varvec{X}} \right)^{ - 1} \varvec{X}^{'} \hat{\varvec{V}}^{ - 1} \varvec{y}$. To obtain the variance of $\hat{\varvec{\beta }}$ and covariance of $\hat{\varvec{\beta }}$ and $\hat{\varvec{\theta }}$, the second order derivatives of (7) are substituted. These are actually the asymptotic variance of $\hat{\varvec{\beta }}$ and covariance of $\hat{\varvec{\beta }}$ and $\hat{\varvec{\theta }}$:

13 $$\frac{{\partial^{2} L_{1} }}{{\partial\varvec{\beta}\partial\varvec{\beta}^{'} }} = - \varvec{X}^{'} \varvec{V}^{ - 1} \varvec{X},$$

14 $$\frac{{\partial^{2} L_{1} }}{{\partial\varvec{\beta}\partial \theta_{l} }} = - \varvec{X}^{'} \varvec{V}^{ - 1} \frac{{\partial \varvec{V}}}{{\partial \theta_{l} }}\varvec{V}^{ - 1} \left( {\varvec{y} - \varvec{X\beta }} \right).$$

Note that in taking expectation of (14), we obtain $E\left( {\partial^{2} L_{1} /\partial\varvec{\beta}\partial \theta_{l} } \right) = 0$ and hence all covariances of $\hat{\beta }_{l}$ and $\hat{\theta }_{{l^{'} }}$ are 0 [19].

## Additional files

10.1186/s13104-016-1912-y A list of the pain intensity data.
10.1186/s13104-016-1912-y Supplemental figures for the pain intensity data (residual plots, P–P plots and Cook’s distance against leverage/(1-leverage)).

## Abbreviations

CV: coefficient of variation
ICC: intra-class correlation coefficient
IRB: institutional review board
LSM: least square mean
MAGE: mean amplitude of glycemic excursion
ML: maximum likelihood
PV: pain vision
REML: restricted maximum likelihood
VAS: visual analog scale
