# Supplementary material for: Comparison of intra-individual coefficients of variation on the paired sampling data when inter-individual variations are different between measures
Source: BMC Res Notes. 2016 Feb 19;9:115. doi: 10.1186/s13104-016-1912-y (PMC4760001; doi:10.1186/s13104-016-1912-y)
Supplement: Supplementary file 2 — 10.1186/s13104-016-1912-y Supplemental figures for the pain intensity data (residual plots, P–P plots and Cook’s distance against leverage/(1-leverage)). [file 13104_2016_1912_MOESM2_ESM.docx]

-2.5

VAS

PV

Standardized residual


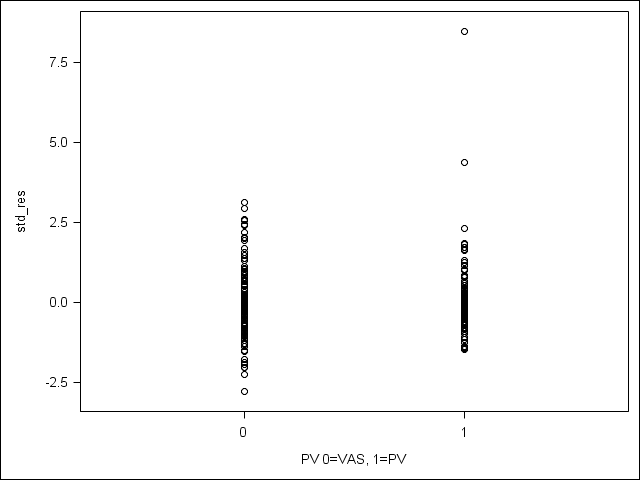


0

2.5

5.0

7.5

Supplemental Figure 1: Residual plots for VAS/PV


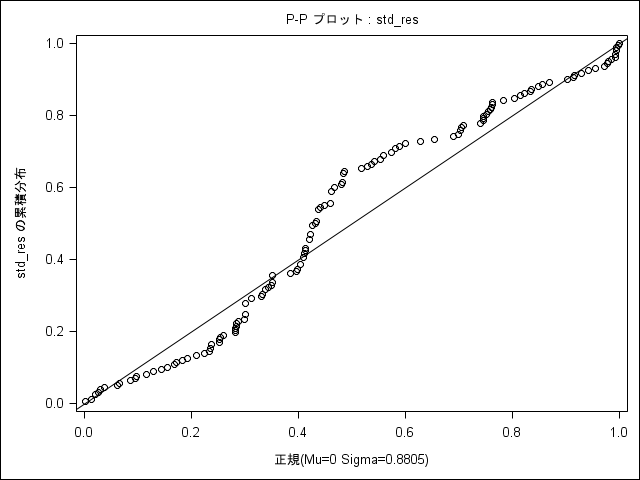

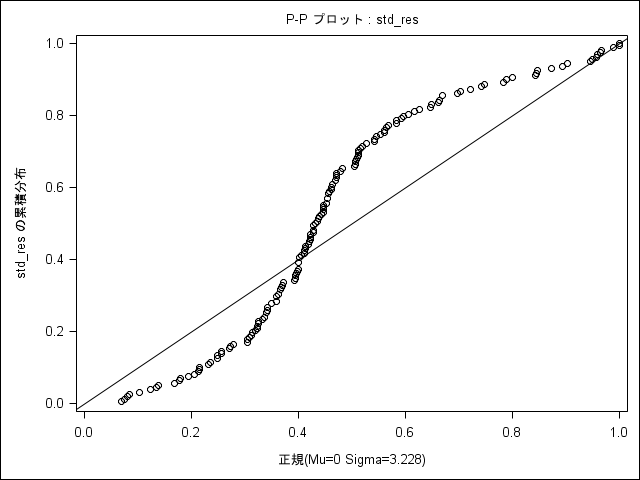


0.0

0.2

0.4

0.6

0.8

1.0

0.0

0.2

0.4

0.6

0.8

1.0

0.0

0.2

0.4

0.6

0.8

1.0

Observed probability

Observed probability

Expected probability

VAS

PV

Supplemental Figure 2: P-P plots by measure


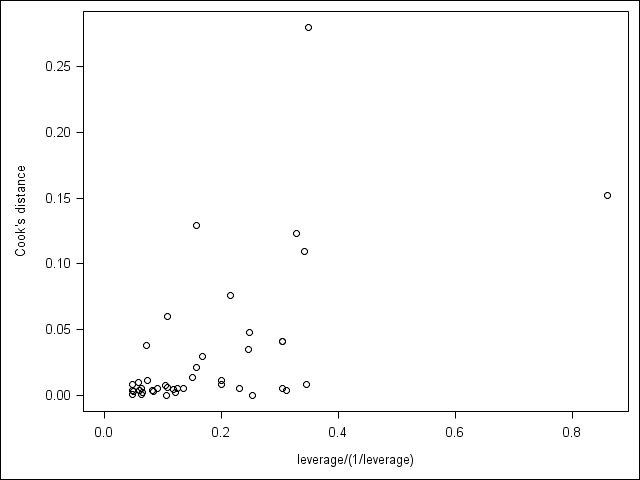


0.0

0.1

0.2

Cook’s distance

leverage/(1-leverage)

0.0

0.2

0.4

0.6

0.8

Supplemental Figure 3: Plot of Cook’s distance vs. Leverage/(1-leverage) to evaluate influential patients

One subject had a very large leverage/(1-leverge) value because she is very much younger than other female subjects. This subject had no impact on CV comparison because the effect of age is not statistically significant.
